# The cost-effectiveness of Cochlear implants in Swedish adults

**DOI:** 10.1186/s12913-021-06271-0

**Published:** 2021-04-08

**Authors:** Mutsa Gumbie, Emma Olin, Bonny Parkinson, Ross Bowman, Henry Cutler

**Affiliations:** Macquarie University Centre for the Health Economy, Level 1, 3 Innovation Rd, Sydney, NSW 2109 Australia

**Keywords:** Cochlear implant, Hearing loss, Costs, Economic evaluation, Quality of life

## Abstract

**Background:**

Research has shown unilateral cochlear implants (CIs) significantly improve clinical outcomes and quality of life in adults. However, only 13% of eligible Swedish adults currently use a unilateral CI. The objective was to estimate the cost-effectiveness of unilateral CIs compared to a hearing aid for Swedish adults with severe to profound hearing loss.

**Methods:**

A Markov model with a lifetime horizon and six-month cycle length was developed to estimate the benefits and costs of unilateral CIs from the Swedish health system perspective. A treatment pathway was developed through consultation with clinical experts to estimate resource use and costs. Unit costs were derived from the Swedish National Board of Health and Welfare and the Swedish Association of Local Authorities and Regions. Health outcomes were reported in terms of Quality Adjusted Life Years (QALYs).

**Results:**

Unilateral CIs for Swedish adults with severe to profound hearing loss are likely to be deemed cost-effective when compared to a hearing aid (SEK 140,474 per QALY gained). The results were most sensitive to the age when patients are implanted with a CI and the proportion of patients eligible for CIs after triage.

**Conclusions:**

An increase in the prevalence of Swedish adults with severe to profound hearing loss is expected as the population ages. Earlier implantation of unilateral CIs improves the cost-effectiveness among people eligible for CIs. Unilateral CIs are an efficacious and cost-effective option to improve hearing and quality of life in Swedish adults with severe to profound hearing loss.

**Supplementary Information:**

The online version contains supplementary material available at 10.1186/s12913-021-06271-0.

## Background

The World Health Organisation estimates that 466 million people worldwide have disabling hearing loss of which 93% are adults and 7 % are children [1]. The number of people with hearing loss in developed countries is set to increase due to population ageing. In Sweden it is estimated that approximately 0.2% of the adult population, or 20,000 people, have severe to profound hearing loss [2].

Hearing loss can reduce a person’s ability to communicate and be socially engaged. It can lead to social stigma, increased anxiety and depression, reduced health related quality of life, and poorer lifetime economic outcomes [3–5]. Cochlear implants (CIs) are a safe and clinically effective intervention for persons with severe to profound hearing loss who gain no satisfactory benefit from using hearing aids. Several studies have found CIs can significantly improve the quality of life of persons with severe to profound hearing loss [6–9].

CIs can improve speech, cognitive function and social interaction [10–12] and can reduce depression and anxiety [13]. CIs also improve quality of life regardless of patients’ age [14] and can also improve the quality of life of family members [13].

CIs are publicly funded in Sweden and patient candidacy criteria for CIs are set by the Swedish National Board of Health and Welfare [15]. To be eligible for a CI a patient must: have severe or profound hearing loss, measured using the Pure Tone Average cut-off threshold of 0.5, 1, 2 and 4 kHz ≥70 dB HL on the better hearing ear; not receive adequate benefit from using acoustic hearing aids, with 50 dB HL or less at 4 kHz, or with a score of 50% or less on monosyllabic-words test; and not have any comorbidities that may affect the intervention.

CIs are underutilised in Sweden, particularly among the older age groups, despite the reported clinical benefits [2]. Approximately 13% (2624 adults) of the estimated eligible adults with severe to profound hearing loss in Sweden use a unilateral CI [16]. Low utilization of CIs in developed countries has been attributed to a range of factors that include a lack of screening for hearing loss in adults, and a lack of awareness of CI candidacy criteria and outcomes among physicians and audiologists [17].

Adults with severe to profound hearing loss may also delay seeking access to a CI because they deny the severity of their hearing loss [18], or may adapt to their condition over time. They may be concerned about the perceived inconvenience of accessing hearing rehabilitation, and the potential costs associated with CIs and their maintenance [18]. Some older adults may place a higher priority on competing comorbidities, imposing another barrier to accessing hearing rehabilitation services [19]. Consequently, adults with severe to profound hearing loss may miss out on potential benefits from CIs [2]. Concerns about risks associated with CI surgery and loss of residual hearing may also reduce the use of unilateral CIs [20].

Reducing the burden of hearing loss is of national importance due to the increased utilisation of healthcare resources and loss of future income associated with hearing loss [21]. Resource allocation decisions impose an opportunity cost because it means other healthcare interventions cannot be funded. Consequently, economic evaluation, as a comparative analysis of intervention alternatives, is used by some governments to help inform which interventions should be prioritised based on a systematic comparison of costs and benefits of interventions for conditions that burden patients and society [22].

Cost utility analyses measure outcomes using Quality Adjusted Life Years (QALY). A QALY is estimated by weighting each life-year with a health state utility score. Health state utility scores are preference-based measures of quality of life using a cardinal scale, where 0 corresponds to death, and 1 to perfect health. Thus, a QALY incorporates quality of life and length of life in one metric, adjusted for preferences for each outcome [23].

Results of a cost utility analysis are presented using the Incremental Cost-Effectiveness Ratio (ICER), which is calculated by taking the incremental cost of the intervention compared to a comparator, divided by the incremental effectiveness of the intervention measured using QALYs [23].

Some European countries use a cost effectiveness threshold to identify pharmaceutical and medical device interventions that represent good value for money [24]. The UK has an explicit cost effectiveness threshold of between £20,000 to £30,000, while the Netherlands has an implicit cost effectiveness threshold [25]. Decision makers are less likely to approve a new pharmaceutical or medical device if its ICER is above the cost effectiveness threshold, although other factors related to cost effectiveness uncertainty, the intervention itself, the targeted population characteristics, and other benefits and costs not captured by the ICER, are also considered by decision makers [24] .

While there is no identified comprehensive health technology assessment (HTA) of CIs in Sweden, the National Board of Health and Welfare published a national indication for unilateral CIs for adults in 2011 [15]. The document included a discussion on the results of an economic evaluation of unilateral CIs in the Swedish setting and reported an estimated ICER of SEK 283,000 (approximately EUR 26,400) per QALY gained for unilateral CIs in Sweden compared to no intervention [15]. The evaluation did not conclude whether unilateral CIs were considered cost effective and no cost effectiveness threshold was discussed. Even though Sweden has relaxed its criteria for CIs since 2011, there is no updated cost effectiveness analysis of unilateral CIs for adults in the Swedish setting [26].

Our study objective was to determine whether unilateral CIs are cost effective compared to hearing aids in Swedish adults with severe to profound hearing loss who previously gained some benefit from using a hearing aid. The model objective was to estimate an ICER using a clinical pathway developed through consultation with two Swedish clinical experts at the two largest CI centres in Sweden and a cost-effectiveness threshold of SEK 250,000 per QALY gained.

## Methods

### Time horizon, discount rate and perspective

The analysis was performed from the Swedish healthcare system perspective, which provides funding for unilateral CIs, over a lifetime horizon to capture all differences in lifetime costs and health outcomes following unilateral CI surgery. As people have a positive rate of time preference, both health benefits and costs arising in the future need to be discounted [23]. Future costs and health outcomes in this analysis were discounted at 3 % per annum in line with the national recommendations in Sweden [27]. No direct or indirect costs borne by the patient were included in the model.

The Dental and Pharmaceutical Benefits Agency, the National Board for Health and Welfare and the Swedish Association of Local Authorities and Regions have not defined a specific cost effectiveness threshold for pharmaceuticals and medical devices in Sweden. However, in the National Guidelines on cardiac care by the National Board for Health and Welfare the cost per QALY has been described using the following categories [28]:

- Low cost per QALY < SEK 100,000
- Moderate cost per QALY is between SEK 100,000 – 499,999
- High cost per QALY > SEK 500,000
- Very high cost per QALY > SEK 1 million.

The moderate cost effectiveness threshold of SEK 250,000 (approximately £20,000) per quality adjusted life year (QALY) gained was used in this study, which lies within the cost-effectiveness threshold range used by the National Institute for Health and Care Excellence (NICE) in the UK.

The model included adults aged 19 years and older with severe to profound hearing loss, with an average age of 61 years. This is the average that adults with severe to profound hearing loss first received a unilateral CI in Sweden, according to the National Board of Health and Welfare [28]. Swedish life tables informed the all-cause mortality rate for the health states [29].

### Model structure

The analysis was performed using a Markov model built in Microsoft Excel®. A Markov model starts with a population of interest, in this case Swedish adults within severe to profound hearing loss. A person occupies a mutually exclusive ‘state’ at each point in time [23, 30]. Death is included as an absorbing state because once a patient dies and has entered the state, they remain there [23, 30]. The model captures the probability of patients remaining in a state or transitioning into another state [23, 30]. These transitions occur within a defined period, called a ‘Markov cycle’. In each Markov cycle, individuals have a certain probability of moving between states, shown by arrows on the Markov model diagram [23, 30].

The Markov model in this study was developed with a six-month cycle length. This was based on the natural history of severe to profound hearing loss, and to capture the impacts from short term adverse events associated with CI surgery. The model compared a unilateral CI to a hearing aid for patients who previously received some benefit from using a hearing aid.

**Fig. 1:**
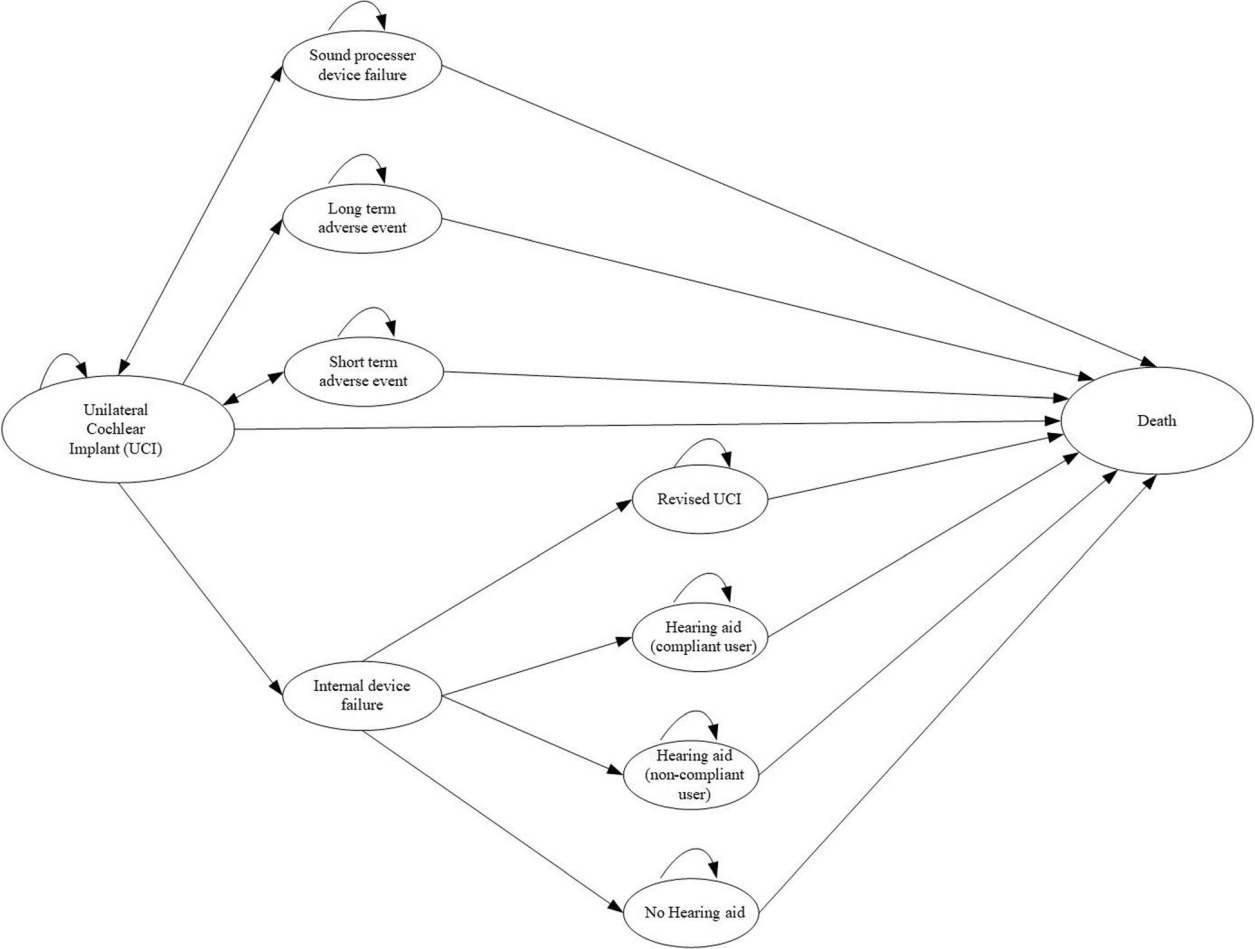
Markov model structure for unilateral cochlear implants

The model incorporated several states to capture the treatment pathway, potential adverse events, internal and sound processer device failures, and death from other causes (Fig. 1). Transition probabilities and inputs for the Markov model structure in Fig. 1 are in Table 1. Table 1 also provides the assumptions applied in the Markov model structure in Fig. 1.

**Table 1:** Model parameters

Event	Parameter	Mean value	95% Confidence interval	Reference
Cochlear implant surgery and hearing aid use	Proportion of people deemed eligible for a cochlear implant after initial assessment	0.56	0.048, 0.985	Expert clinical opinion
	Death from cochlear implant surgery	0.00	–	Assumption
	Probability of internal device failure having an implant revision surgery	1.0	–	Assumption
	Probability a patient elects to discontinue using their cochlear implant	0.077	0.009, 0.206	[31]
	Proportion of people who receive a benefit from using a hearing aid	0.50	0.061, 0.939	[5]
	Proportion of unilateral candidates adopting a hearing aid and are compliant	0.50	0.061, 0.939	[5]
Device failure	Risk of a cochlear implant internal failure^1^	0.025	0.011, 0.040	[32]
	Risk of a cochlear implant external (sound processor) failure^1^	0.004	0.002, 0.018	
	Six-month probability of internal or external device failure	0.006	–	Calculated from [32]. Assumed time to external and internal device failure was similar.
Adverse events	Dysgeusia (taste disturbance) (short term)	0.065	–	Weighted average of several papers [33–38]
	Vertigo (short term)	0.082	–	
	Tinnitus (short term)	0.039	–	
	Wound infection (short term)	0.045	–	
	Vertigo (long term)	0.014	–	
Device upgrading	Mean lifetime of an acoustic hearing aid	5 years	1.3 years, 11 years	[5]
	Mean time to sound processor upgrade	Every 106 months	29 months, 232 months	Cochlear Limited

Note: 1. Failure rates were derived from a retrospective review of 235 cases of cochlear implant revisions between 1982 and 2011 within the Sydney (Australia) Cochlear Implant Centre [32]. While internal data from Cochlear Limited suggested lower failure rates are associated with Cochlear Limited implants, these may not represent the average failure rate of all manufacturer’s cochlear implants available in Sweden

Patients can remain in their initial state or experience a short term adverse event or long term adverse event. If there is an internal device failure, the patient may receive a revised CI, or use a hearing aid, or not use a hearing aid.

Consequently, patients in the ‘Unilateral cochlear implant (UCI)’ state may either:

- remain in the ‘UCI’ state;
- experience a long term adverse event (i.e., transition to ‘Long term adverse event’ state);
- experience a sound processer device or internal device failure (i.e., transition to ‘External device failure’ or ‘Internal device failure’ states);
- have their CI revised (i.e., transition to ‘Revised UCI’ state);
- voluntarily or non-voluntarily stop using CIs (i.e., transition to ‘Hearing aid (compliant user)’ or ‘Hearing aid (non-compliant user)’ or ‘No hearing aid’ states); or
- die from surgery or natural causes (i.e., transition to ‘Death’ state).

**Fig. 2:**
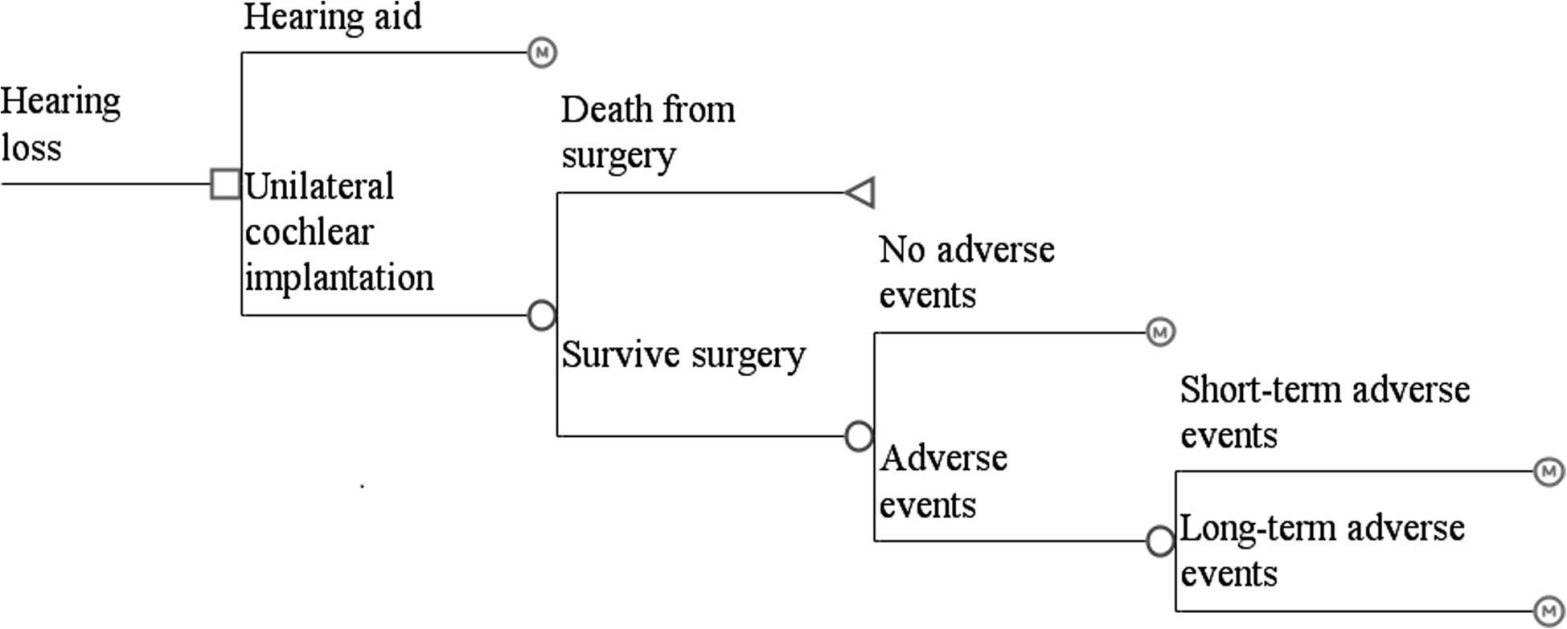
Treatment pathway for adults with severe to profound sensorineural hearing loss

Adverse events can occur to patients who receive a CI. These were categorised as either short term adverse events that lasted one cycle (i.e., 6 months), or long-term events that lasted for the patient’s lifetime (Fig. 2).

**Fig. 3:**
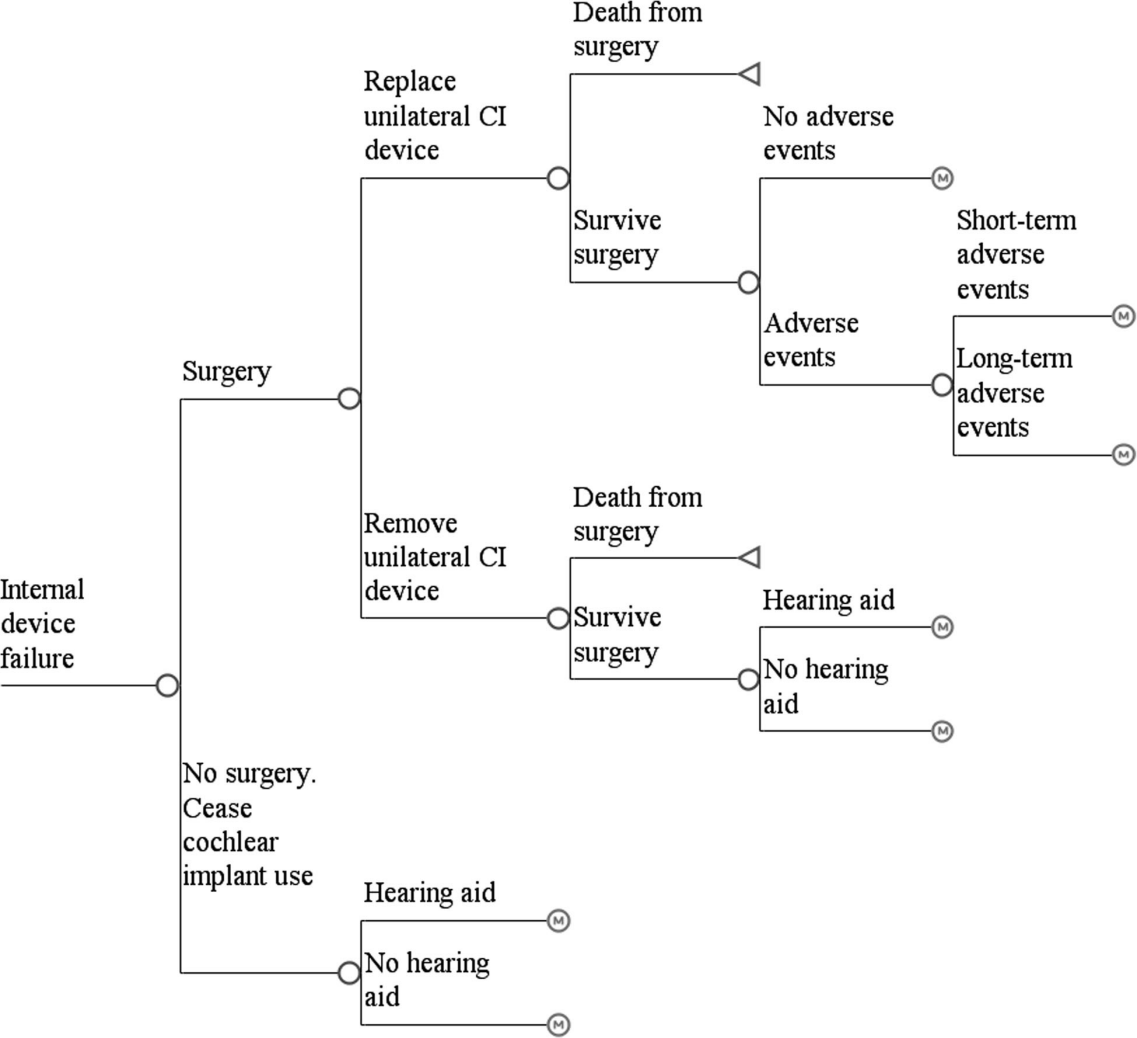
Treatment pathway for patients experiencing and internal device failure

Internal and sound processor device failure or sound processor upgrade can either be immediate (in the first cycle) following surgery, or they can occur in the future. This was incorporated into the model using a time-dependent probability calculated from cumulative survival values. Patients who experience an internal device failure may choose to re-operate and replace the device (Fig. 3). These patients may experience an adverse event, which could either be short term or long term. Patients who experience an internal device failure may also choose to re-operate and remove the device permanently. Alternatively, they could remain with a faulty device, but they cease to receive the benefits from the CI.

Patients who experience a sound processor device failure may choose to replace the device or remain with a faulty device but they cease to receive the benefits from the CI. There is no risk of death or adverse events with sound processor device failure.

‘Death’ is an absorbing state, accounting for the risk associated with a unilateral CI surgery and general all-cause mortality over time in the Swedish adult population.

### Model assumptions

Several assumptions were required to be made in the model, which were validated by either the literature or clinical experts [39, 40]. The model assumed that there was no risk of death or adverse events with internal device failure if there is no surgery to remove the CI, as there is no evidence of any increased probability of death or severe adverse events associated with using a CI. Patients are assumed to receive a sound processor upgrade every 8.83 years, which is the general trend [41]. The model assumed that unilateral CI processor upgrade does not provide additional health related quality of life benefits. It was also assumed that 50% of all wound infections resulting from surgery requires an explant, antibiotic use, and re-implantation, all occurring within 6 months [39].

### Model inputs

An extensive search of the literature was undertaken to populate the Markov model. Expert opinion from two clinicians was used to fill data gaps.

#### Measure of effectiveness: utilities

Improvements in utilities from unilateral CIs were based on a prospective longitudinal study conducted in Sweden, on 38 adults with profound hearing loss [42]. The study reported utility values before and after receiving a unilateral CI. The utility increment from receiving a unilateral CI was assumed to be uniform across age.

Utility decrements from severe to profound sensorineural hearing loss were calculated by subtracting the utility value of having no unilateral CI or hearing aid, from the Canadian HUI3 population utility norm.^1^ This allowed the model to reduce population norm utilities over time, reflecting the poorer quality of life associated with comorbidities and ageing (Table 2).

**Table 2:** Utility values

Health state	Utility	Reference
**Severe and profound hearing loss prior to a cochlear implant**		
Eligible CI candidates^1^	0.450	[42]
**Utility decrement from population utility norms for persons with severe and profound hearing loss**		
Eligible candidates	0.391	Calculated by subtracting the HUI3 utility score of having no cochlear intervention from the Canadian HUI3 population utility norm for people 61 years of age.
**Utility increment associated with receiving a unilateral cochlear implant**		
Eligible candidates	0.210	[42]

Note: 1. Patients for whom previous hearing aid use provided some benefit. Utility scores were for Swedish patients with an average age of 71 years, one year-post implantation and the model assumed these values to be similar for patients aged 61 years. Abbreviation: CI – cochlear implant

#### Utility decrements associated with adverse events

A literature review was conducted to identify short term adverse events and long term adverse events associated with CI surgery and their utility decrements. There were 17 types of adverse events associated with CIs found within the literature, excluding device complications such as electrode migration. These were allocated to six categories, including infection, neurological complications, pain, tinnitus, vestibular complications and other complications.

The probability of receiving many types of adverse events was small, with most equating to less than 1 %. Only adverse events with a prevalence greater than 1 % were included in the model. These were three short term adverse events (taste disturbances, vertigo, and infection) and one long term adverse event (tinnitus).

It was assumed that short term adverse events would last for 6 months. Adverse events were included in the model as a weighted average of their associated disutilities (Table 3), using the probability of experiencing the adverse event as their weight. It was assumed that 50% of wound infections require an explantation, antibiotic use, and re-implantation, all occurring within 6 months. Utility decrement associated with tinnitus were assumed to last a lifetime. Health outcomes were reported in QALYs.

**Table 3:** Disutility values

Health state	Disutility	Duration	Reference
**Short term adverse events**			
Dysgeusia (taste disturbance)	0.020	6 months	Model assumption
Vertigo	0.033	6 months	[43]
Tinnitus	0.050	6 months	[44]
Wound infection	0.042	6 months	[45]
**Long term adverse events**			
Vertigo	0.033	Lifetime	[43]

#### Resource use

**Fig. 4:**
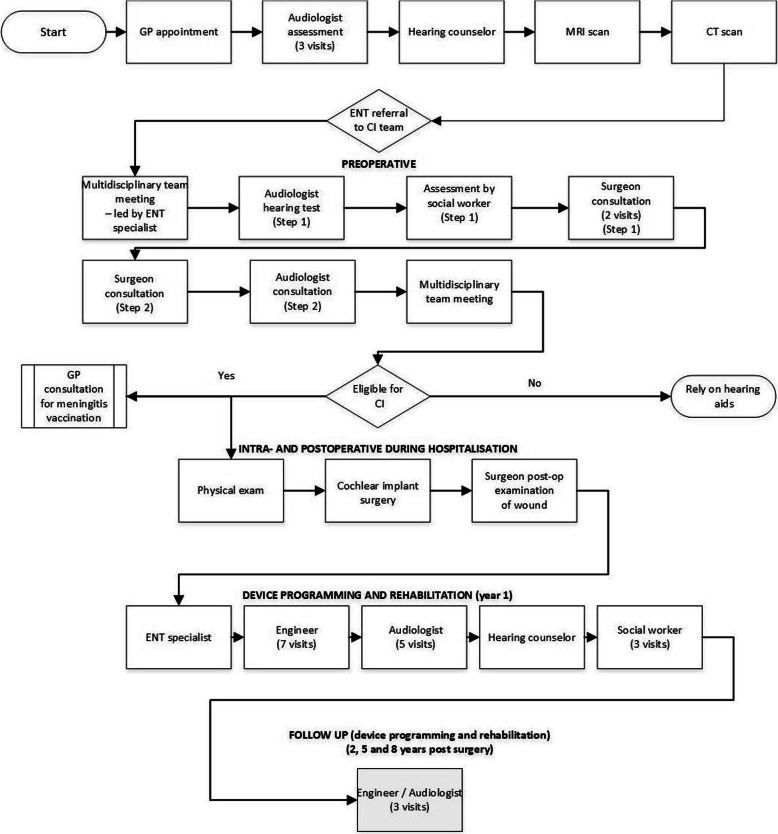
Pathway for resource use associated with a cochlear implant. Note: Does not include resource use associated with sound processor replacement, explantation and re-implantation and adverse events. Abbreviations: CI = Cochlear Implant, CT = computed tomography, ENT = Ear, Nose and Throat; GP = general practitioner, MRI = magnetic resonance imaging, SLT = Speech and Language Therapist

Resource use was derived from a patient pathway developed from the expert opinion of two Swedish medical practitioners’. The patient pathway represented the types of resources an average patient is expected to use, from receiving an initial referral to a CI program from an audiologist to ongoing use of a unilateral CI. The patient pathway included two preoperative evaluation steps following an initial referral to a CI team before patients could be deemed eligible for a unilateral CI (Fig. 4).

Resource use was divided into four categories within the economic evaluation model, including:

- pre-implant assessment;
- surgery (the CI device and surgery);
- device programming and maintenance; and
- complications (short and long term adverse events, sound processor device or internal device failure).

#### Unit costs

Assumptions related to resource use and cost were sought through expert opinion from two clinicians and used within the model [39, 40]. These included:

- Patients entering a CI program for the first time receive a referral from their ear, nose and throat specialist;
- Patients undergoing an audiological assessment for unilateral CIs have both ears investigated within one assessment;
- Approximately 56% of patients referred for a unilateral CI receive a unilateral CI after initial evaluations; and
- Annual equipment maintenance costs start in the second year from receiving a unilateral CI.

Unit costs were derived from the Swedish National Board of Health and Welfare, Swedish Association of Local Authorities and Regions and clinical expert opinion. All costs were expressed in 2019 SEK prices. Total costs were calculated by multiplying the volume of resource use by their unit cost (Additional file 1).

### Sensitivity and scenario analyses

The results were summarised using an ICER, calculated by dividing the difference in total costs (incremental cost) by the difference in the QALYs (incremental effect).

The ICER was compared to a cost-effectiveness threshold of SEK 250,000 (approximately EUR 23,000) per QALY gained. This lies within the cost-effectiveness threshold range used by the National Institute for Health and Care Excellence (NICE) in the UK [46].

Sensitivity analysis was undertaken to assess the sensitivity of the ICER to key model parameters. Parameters were chosen based on their expected level of uncertainty and potential influence on the ICER. Their range was dictated by the 95% confidence interval around the estimate (where available), while ranges based on plausible values were determined for all other parameters.

A deterministic sensitivity analysis was undertaken that included manually changing key model parameters individually and collectively. This provided some insight into the sensitivity of the ICER to specific parameters of interest expected to have a large impact (e.g., the cost of a unilateral CI), highlighting the importance of key parameters within the model.

Probabilistic sensitivity analysis using Monte Carlo simulation was also conducted, using distributions attached to each model parameter, with 10,000 samples drawn at random from these distributions to calculate an ICER distribution. A cost-effectiveness acceptability curve (CEAC) was produced to summarise the impact of uncertainty on the ICER being cost-effective [47]. The CEAC indicates the probability that an intervention is cost-effective when compared with the alternative, for a range of values of cost-effectiveness thresholds [48].

Three scenarios analyses were undertaken to explore the impact on the ICER: 1) lowering the average age of patients receiving a unilateral CI from 61 years to 50 years; 2) decreasing the duration between processor upgrades from every 8.83 years to every 5 years, and; and 3) increasing the proportion of patients eligible after triage from 56% to 70%.

## Results

### Base case results

It was estimated that a unilateral CI compared to a hearing aid for Swedish adults with severe to profound hearing loss who previously gained some benefit from using a hearing aid increased health costs by SEK 435,147, and increased health-related quality of life by 3.10 QALYs, on average. This equates to an ICER of SEK 140,474 per QALY gained (Table 4).

**Table 4:** Cost-effectiveness results

	Comparator Hearing aid		Intervention Unilateral Cochlear Implant	
	Cost (SEK)	QALY	Cost (SEK)	QALY
Base Case				
**Mean Values**	7776	5.74	442,923	8.84
**Incremental Values**	–		435,147	3.10
**ICER (Cost/QALY)**	–		140,474	
Scenario Analysis – Lowering the Average Starting age from 61 years to 50 years				
**Mean Values**	11,072	8.01	500,376	12.15
**Incremental Values**	–		489,304	4.14
**ICER (Cost/QALY)**	–		118,232	
Scenario Analysis – Increasing the Frequency of Processor Upgrade from every 9 years to every 5 years				
**Mean Values**	7776	5.74	513,223	8.84
**Incremental Values**	–		505,447	3.10
**ICER (Cost/QALY)**	–		163,169	
Scenario Analysis – Increasing the Proportion eligible after triage from 56 to 70%				
**Mean Values**	7776	5.74	432,048	8.84
**Incremental Values**	–		424,272	3.10
**ICER (Cost/QALY)**	–		136,964	
Scenario Analysis – Lower age, Increasing Upgrade Frequency and Increasing the Proportion eligible after triage				
**Mean Values**	11,072	8.01	585,296	12.15
**Incremental Values**	–		574,224	4.14
**ICER (Cost/QALY)**	–		138,751	

Abbreviations: *ICER* incremental cost-effectiveness ratio, *QALY* quality adjusted life years

### Scenario analysis

#### Patient age

It was estimated that lowering the patient age from 61 to 50 years in the model reduced the ICER associated with a unilateral CI to SEK 118,232 per QALY gained (Table 4).

#### Frequency of sound processor device upgrade

It was estimated that increasing the frequency of sound processor upgrade from every 9 years to every 5 years increased the ICER associated with a unilateral CI to SEK 163,169 per QALY gained (Table 4).

#### Proportion eligible after triage

It was estimated that increasing the proportion of patients eligible after triage from 56% to 70% reduced the ICER associated with a unilateral CI to SEK 136,964 per QALY gained (Table 4).

#### Multiway scenario analysis

It was estimated that lowering the starting age to 50 years, increasing the frequency of processor upgrade to every 5 years, and increasing the proportion eligible after triage to 70% decreased the ICER associated with a unilateral CI to SEK 138,751 per QALY gained (Table 4).

### Sensitivity analysis

**Fig. 5:**
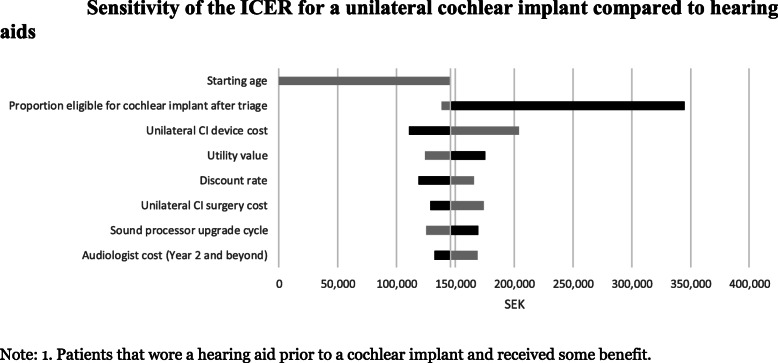
Sensitivity of the ICER for a unilateral cochlear implant compared to hearing aids. Note: 1. Patients that wore a hearing aid prior to a cochlear implant and received some benefit

The ICER associated with a unilateral CI compared to a hearing aid for patients who previously gained some benefit from using a hearing aid was most sensitive to the starting age; the proportion of people eligible for a unilateral CI after triage; the utility increment associated with a unilateral CI for hearing aid users; and the cost of the CI device (Fig. 5).

**Fig. 6:**
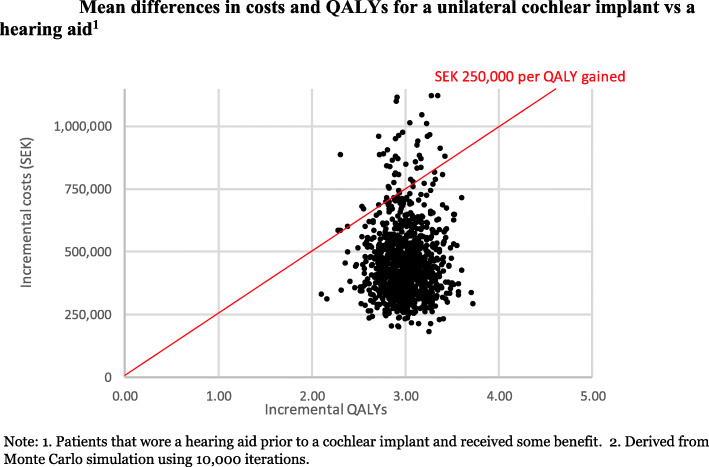
Mean differences in costs and QALYs for a unilateral cochlear implant vs a hearing aid^1.^ Note: 1. Patients that wore a hearing aid prior to a cochlear implant and received some benefit. 2. Derived from Monte Carlo simulation using 10,000 iterations

**Fig. 7:**
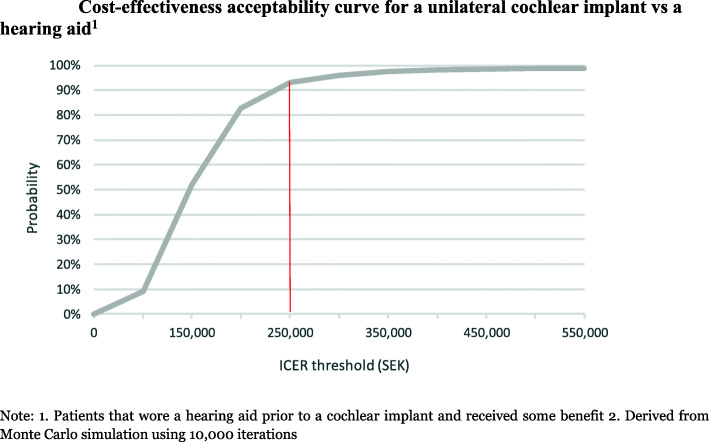
Cost-effectiveness acceptability curve for a unilateral cochlear implant vs a hearing aid^1^. Note: 1. Patients that wore a hearing aid prior to a cochlear implant and received some benefit 2. Derived from Monte Carlo simulation using 10,000 iterations

All simulated results for a unilateral CI fall into the north-east quadrant of the cost-effectiveness plane, indicating that a unilateral CI compared to a hearing aid is more expensive but also more effective (Fig. 6). Most of the simulated mean differences in costs and QALYs were below the SEK 250,000 per QALY gained threshold. Overall it was estimated that a unilateral CI has a 92% likelihood of being cost-effective compared to using a hearing aid with some benefit (Fig. 7). This is equivalent to stating that, given the data, there is a 92% chance that a unilateral CI, compared with using a hearing aid, is at or below SEK 250,000 per QALY gained. When a cost-effectiveness threshold of SEK 100,000 per QALY was used, the CEAC shows that a unilateral CI has a 9 % likelihood of being cost-effective when compared to a hearing aid.

When a cost-effectiveness threshold of SEK 500,000 per QALY was used, the CEAC shows that a unilateral CI has a 99% likelihood of being cost-effective when compared to a hearing aid.

## Discussion

This model was developed to assess the cost-effectiveness of a unilateral CI from a Swedish healthcare payer perspective for the treatment of severe to profound hearing loss in adult patients when, at the point of treatment initiation, they derived some benefit from hearing aids. With an estimated ICER of SEK 140,474 per QALY, unilateral CIs for Swedish adults with severe to profound hearing loss are likely to be considered cost-effective by decision-makers when compared to a hearing aid, using a cost-effectiveness threshold of SEK 250,000 per QALY gained.

Our results are similar to the Health Technology Assessment report on CIs in adults in the United Kingdom conducted on behalf of NICE in 2009 [49]. It found the ICER for unilateral CIs compared with no implantation was £14,163 (approximately SEK 165,000) per QALY for patients aged 50 years, with a 100% probability of being cost-effective at a £20,000 (approximately SEK 250,000) per QALY threshold.

There are some important differences between this study and the NICE HTA report. The proportion of people eligible for a CI after triage was much lower in this study (56%), compared to the NICE HTA report (70%). Differences in methodology and assumptions will also impact the results. For example, this study had an average age of 61 years for receiving a unilateral CI, whereas patients in the NICE HTA report had an average age of 50 years. The NICE HTA report assumed reduced costs for internal device failure, repairs and replacement within a warranty period, which this study did not. Other important differences between the two studies include different unit costs, different adverse events resulting from a CI, and different Markov model structures.

Our estimated ICER is almost half the estimated ICER of SEK 283,000 (approximately EUR 26,400) per QALY gained for unilateral CIs compared to no intervention reported by the National Board of Health and Welfare for Swedish adults in 2011 [15]. However, unilateral CI pathways and costs are likely to have changed in Sweden over the past decade [15].

Sensitivity and scenario analyses indicated that the ICER was most sensitive to the starting age and the proportion of people eligible for CIs. Results from the scenario analysis indicated that lowering the model starting age of patients receiving a unilateral CI in Sweden from 61 years to 50 years reduced the ICER from SEK 140,474 to SEK 118,232 per QALY gained.

This suggests earlier implantation of unilateral CIs in eligible patients could improve cost-effectiveness.

The ICER within this study indicates that unilateral CIs are potentially more cost-effective compared to some common medical interventions for adults in Sweden. For example, the cost per QALY gained for a unilateral CI is lower than that for knee replacement surgery (SEK 150,454 per QALY gained), unilateral hip replacement surgery (SEK 337,083 per QALY gained) and prostheses for patients with transfemoral amputation (SEK 868,479 per QALY gained) [50–52] (See Table 5). However, differences in these ICERs may also reflect differences in study methodologies.

**Table 5:** Cost-effectiveness of established medical procedures versus unilateral cochlear implantation

Surgical procedure	SEK/QALY	Reference
Shunt surgery for idiopathic normal pressure hydrocephalus	80,600	[53]
Unilateral cochlear implantation	140,474	Study results
Knee replacement	150,454	[52]
Flash Glucose Monitoring System for Patients with Type 1 Diabetes Receiving Intensive Insulin Treatment	291,130	[54]
Unilateral hip replacement	337,083	[50]
Transfemoral amputation	868,479	[51]

The increasing prevalence of hearing loss due to an ageing population in Sweden will increase demand for unilateral CIs in the future. The Swedish government will be under pressure to provide greater access to CIs, which will place additional pressure on healthcare budgets. Some pressure can be relieved by reallocating healthcare resources from other devices and services, although choosing which ones to defund is complex.

Despite the likelihood of cochlear impacts being cost effective in Sweden, many people who could benefit from a unilateral CI have not received one [2, 16]. CIs are a voluntary (elective) therapy that requires a patient to choose to receive the implant. Other implants, such as pacemakers and shunts, are less voluntary and are typically implanted based on a specialist decision at the time of an emergency.

Implantable medical devices, such as knee and hip replacements, are also elective therapies. However, eligibility is primarily determined by the specialist, who is incentivised to induce demand [55]. In contrast, access to CIs is based on candidacy criteria set by the Swedish government. Applying the less restrictive CI criteria, introduced in the United Kingdom and the Dutch-speaking areas of Belgium, to a CI patient referral centre in the Netherlands resulted in a 30% increase of patients implanted with CIs [56]. Relaxing cochlear candidacy criteria in Sweden is expected to increase the number of implanted patients and therefore provide additional health benefits to Swedish adults with severe to profound hearing loss.

### Study strengths and limitations

A major strength of this study is the patient pathway developed through consultation with two Swedish clinical experts at the two largest CI centres in Sweden [39, 40]. Resource use and unit costs were largely based on the clinical experts’ opinion sought within the development of the patient pathway. This is the first time a patient pathway has been explicitly developed for unilateral CIs in Sweden to assess their cost-effectiveness. It has allowed the model to capture the complexity associated with gaining access to a unilateral CI in Sweden and to reflect contemporary resource use and costs. The clinical experts provided the parameter input for the proportion of patients eligible for unilateral CI after triage. The model structure and assumptions were also reviewed and verified by the clinical experts.

One major study limitation is that the utility increment derived from a unilateral CI was based on one prospective longitudinal study of Swedish adults with no control group. The study participants averaged 71 years old, which was ten years older than the starting age used in the model. However, the difference in utility increments for a 61 year old compared to a 71 year old with the same hearing loss from receiving a unilateral CI would be negligible. The average utility increment used in this study also aligns with other studies that have quantified the utility gained from a unilateral CI [5, 57]. Another limitation is that there is some controversy about governments using cost effectiveness thresholds to make decisions on whether to fund a new healthcare intervention, and there is no explicit cost-effectiveness threshold used in Sweden. It is therefore not clear whether the Swedish government would consider a unilateral CI cost effective using the cost effectiveness threshold employed within this study.

## Conclusions

An increase in people with permanent hearing loss is expected with an ageing population. This study has found that a unilateral CI is a cost-effective option to improve hearing in Swedish adults with severe to profound hearing loss who gain some benefit from hearing aids. It also found that earlier implantation of unilateral CIs improves the cost-effectiveness, as does improved identification of people eligible for CIs.

## Supplementary Information

**Additional file 1.** Resource use and unit costs

## Data Availability

Not applicable.

## Abbreviations

CEAC: Cost-effectiveness acceptability curve
CI: Cochlear implant
dB: Decibels in hearing level
EUR: Euro
ICER: Incremental cost-effectiveness ratio
kHz: kiloHertz
NICE: National Institute for Health and Care Excellence
QALY: Quality-adjusted life year
SEK: Swedish Krona
WHO: World Health Organisation

## Footnotes

1 Due to the lack of HUI3 population utility norms for Sweden, the model applied the Canadian set of population utility norms given it was considered the most comprehensive and recent.
