# Supplementary material for: The cost-effectiveness of Cochlear implants in Swedish adults
Source: BMC Health Serv Res. 2021 Apr 8;21:319. doi: 10.1186/s12913-021-06271-0 (PMC8034197; doi:10.1186/s12913-021-06271-0)
Supplement: Supplementary file 1 — Additional file 1. Resource use and unit costs [file 12913_2021_6271_MOESM1_ESM.docx]

**Additional File 1**

**Table 1: Pre-implant assessment resource use and unit costs**

| **Resource** | **No. of visits** | **Units** | **Unit type** | **Unit cost (SEK)** | **Reference** |
| --- | --- | --- | --- | --- | --- |
| **Initial assessments** | | | | | |
| Medical doctor appointment | 1 | 1 | Consultation | SEK 1,406 | Swedish Association of Local Authorities and Regions (Sveriges Kommuner och Landsting (Swedish Association of Local Authorities and Regions), 2019) |
| Fitting of hearing aid | 1 | 1 | Consultation | SEK 1,704 | NordDRG 2019 (Socialstyrelsen (The National Board of Health and Welfare), 2018) |
| Audiological Assessment: Tone / Speech audiogram with / without HA | 1 | 1 | Consultation | SEK 1,476 | NordDRG 2019 (Socialstyrelsen (The National Board of Health and Welfare), 2018) |
| Vestibular assessment and tests: VNG Videonystagmography: Balance investigation / Vestibular assessment and tests | 1 | 1 | Consultation | SEK 1,476 | NordDRG 2019 (Socialstyrelsen (The National Board of Health and Welfare), 2018) |
| Hearing counsellor | 1 | 1 | Consultation | SEK 2,215 | NordDRG 2019 (Socialstyrelsen (The National Board of Health and Welfare), 2018) |
| MRI Scan | 1 | 1 | Test | SEK 2,328 | NordDRG 2019 (Socialstyrelsen (The National Board of Health and Welfare), 2018) |
| CT Scan | 1 | 1 | Test | SEK 2,328 | NordDRG 2019 (Socialstyrelsen (The National Board of Health and Welfare), 2018) |
| **Step 1** | | | | | |
| Multidisciplinary team meeting | 1 | 1 | Consultation | SEK 3,237 | NordDRG 2019 (Socialstyrelsen (The National Board of Health and Welfare), 2018) |
| Audiologist | 1 | 1 | Consultation | SEK 1,476 | NordDRG 2019 (Socialstyrelsen (The National Board of Health and Welfare), 2018) |
| ENT surgeon consultation | 1 | 1 | Consultation | SEK 2,612 | NordDRG 2019 (Socialstyrelsen (The National Board of Health and Welfare), 2018) |
| Social worker assessment | 1 | 1 | Consultation | SEK 2,215 | NordDRG 2019 (Socialstyrelsen (The National Board of Health and Welfare), 2018) |
| **Step 2** | | | | | |
| ENT surgeon consultation | 1 | 1 | Consultation | SEK 2,612 | NordDRG 2019 (Socialstyrelsen (The National Board of Health and Welfare), 2018) |
| Audiologist | 1 | 1 | Consultation | SEK 2,215 | NordDRG 2019 (Socialstyrelsen (The National Board of Health and Welfare), 2018) |
| Multidisciplinary team meeting | 1 | 1 | Consultation | SEK 3,237 | NordDRG 2019 (Socialstyrelsen (The National Board of Health and Welfare), 2018) |
| Meningitis vaccination | 1 | 1 | Consultation | SEK 1,406 | Swedish Association of Local Authorities and Regions (Sveriges Kommuner och Landsting (Swedish Association of Local Authorities and Regions), 2019) |

Abbreviations: CT= Computerized Tomography; ENT= Ear, Nose and Throat; MRI= Magnetic Resonance Imaging.

**Table 2: Surgery, device and device programming and rehabilitation resource use and unit costs**

| **Resource** | **No. of visits** | **Units** | **Unit type** | **Unit cost (SEK)** | **Reference** |
| --- | --- | --- | --- | --- | --- |
| Unilateral CI operation – Surgical cost | 1 | 1 | Visit | SEK 69,744 | Calculated based on NordDRG 2019 (Socialstyrelsen (The National Board of Health and Welfare), 2019) and excluding device cost. |
| Unilateral CI operation – Device cost | 1 | 1 | Unit | SEK 145,000 | Cochlear Nordic AB |
| One hearing aid | 1 | 1 | Unit | SEK 3,404 | Cochlear Nordic AB |
| Pair of hearing aids | 1 | 1 | Unit | SEK 6,807 | Cochlear Nordic AB |
| **Initial care – Year 1** | | | | | |
| Medical check | 1 | 1 | Consultation | SEK 1,406 | Swedish Association of Local Authorities and Regions (Sveriges Kommuner och Landsting (Swedish Association of Local Authorities and Regions), 2019) |
| Audiologist | 5 | 1 | Consultation | SEK 1,476 | NordDRG 2019 (Socialstyrelsen (The National Board of Health and Welfare), 2018) |
| Engineer | 7 | 1 | Consultation | SEK 1,987 | NordDRG 2019 (Socialstyrelsen (The National Board of Health and Welfare), 2018) |
| ENT specialist | 1 | 1 | Consultation | SEK 2,612 | NordDRG 2019 (Socialstyrelsen (The National Board of Health and Welfare), 2018) |
| Hearing counsellor | 1 | 1 | Consultation | SEK 2,2145 | NordDRG 2019 (Socialstyrelsen (The National Board of Health and Welfare), 2018) |
| Social worker | 3 | 1 | Consultation | SEK 2,215 | NordDRG 2019 (Socialstyrelsen (The National Board of Health and Welfare), 2018) |
| **Maintenance and programming, cochlear implant (Year 2 and beyond)** | | | | | |
| Processor Upgrade |  | 1 | Unit | SEK 60,000 | Cochlear Nordic AB |
| Audiologist (Year 2 and beyond) | 3 | 1 | Consultation | SEK 1,476 | NordDRG 2019 (Socialstyrelsen (The National Board of Health and Welfare), 2018) |
| CI annual administration |  | 1 | Unit | SEK 520 | Expert opinion |

Abbreviations: CI= Cochlear Implant; ENT= Ear, Nose and Throat.

**Table 3: Sound processor replacement, adverse events, explantation and re-implantation resource use and unit costs**

| **Resource** | **No. of visits** | **Units** | **Unit type** | **Unit cost (SEK)** | **Reference** |
| --- | --- | --- | --- | --- | --- |
| **Sound processor replacement** | | | | | |
| External component | 1 | 1 | Unit | SEK 60,000 | Cochlear Nordic AB |
| Audiologist (tuning visit) | 1 | 1.5 | Hours | SEK 1,476 | NordDRG 2019 (Socialstyrelsen (The National Board of Health and Welfare), 2018) |
| Engineer (tuning visit) | 1 | 1.5 | Hours | SEK 1,987 | NordDRG 2019 (Socialstyrelsen (The National Board of Health and Welfare), 2018) |
| **Short term adverse event: Taste disturbances, Vertigo, Tinnitus** | | | | | |
| Medical doctor visit | 1 | 1 | Consultation | SEK 1,406 | Swedish Association of Local Authorities and Regions (Sveriges Kommuner och Landsting (Swedish Association of Local Authorities and Regions), 2019) |
| **Infection** | | | | | |
| Medical doctor visit | 1 | 1 | Consultation | SEK 1,406 | Swedish Association of Local Authorities and Regions (Sveriges Kommuner och Landsting (Swedish Association of Local Authorities and Regions), 2019) |
| Surgery | 1 | 1 | Procedure | SEK 276,227 | Model assumption: explantation + reimplantation costs. Also, new implant but no new processor. |
| **Long term adverse event: Vertigo** | | | | | |
| Medical doctor visit | 1 | 1 | Consultation | SEK 1,406 | Swedish Association of Local Authorities and Regions (Sveriges Kommuner och Landsting (Swedish Association of Local Authorities and Regions), 2019) |
| **Explantation** | | | | | |
| Audiologist (assessment) | 1 | 1.5 | Hours | SEK 1,476 | NordDRG 2019 (Socialstyrelsen (The National Board of Health and Welfare), 2018) |
| Explantation | 1 | 1 | Visit | SEK 55,614 | Assumed value to be equivalent to unilateral CI surgery costs |
| **Re-implantation** | | | | | |
| Audiologist pre-operative assessment | 1 | 1.5 | Hours | SEK 1,476 | NordDRG 2019 (Socialstyrelsen (The National Board of Health and Welfare), 2018) |
| ENT surgeon consultation | 1 | 1 | Hours | SEK 2,612 | NordDRG 2019 (Socialstyrelsen (The National Board of Health and Welfare), 2018) |
| Multidisciplinary team meeting  - Audiology - ENT  - Engineer | 1 | 1 | Hours | SEK 3,237 | NordDRG 2019 (Socialstyrelsen (The National Board of Health and Welfare), 2018) |
| Unilateral CI operation – Surgical cost | 1 | 1 | Visit | SEK 55,613.6 | Assumed value to be equivalent to unilateral CI surgery costs |
| Medical check | 1 | 1 | Consultation | SEK 1,406 | Swedish Association of Local Authorities and Regions (Sveriges Kommuner och Landsting (Swedish Association of Local Authorities and Regions), 2019) |

Abbreviations: CI= Cochlear Implant; ENT= Ear, Nose and Throat.

Note: Short term adverse events are defined as those lasting six months or less. Long term adverse events are defined as those lasting a lifetime.
